# Validation and Characterization of a Seed Number Per Silique Quantitative Trait Locus *qSN.A7* in Rapeseed (*Brassica napus* L.)

**DOI:** 10.3389/fpls.2020.00068

**Published:** 2020-02-21

**Authors:** Yaoyao Zhu, Jiang Ye, Jiepeng Zhan, Xiaoxiao Zheng, Jiangjiang Zhang, Jiaqin Shi, Xinfa Wang, Guihua Liu, Hanzhong Wang

**Affiliations:** Key Laboratory of Biology and Genetic Improvement of Oil Crops, Ministryof Agriculture and Rural Affairs, Oil Crops Research Institute of the Chinese Academy of Agricultural Sciences, Wuhan, China,

**Keywords:** *Brassica napus* L., quantitative trait locus, seed number per silique, fine-mapping, cytological mechanism

## Abstract

Seed number is a key character/trait tightly related to the plant fitness/evolution and crop domestication/improvement. The seed number per silique (SNPS) shows a huge variation from several to more than 30, however the underlying regulatory mechanisms are poorly known, which has hindered its improvement. To answer this question, several representative lines with extreme SNPS were previously subjected to systematic genetic and cytological analyses. The results showed that the natural variation of seed number per silique is mainly controlled by maternal and embryonic genotype, which are co-determined by ovule number per ovary, fertile ovule ratio, ovule fertilization rate, and fertilized ovule development rate. More importantly, we also mapped two repeatable quantitative trait loci (QTLs) for SNPS using the F_2:3_ population derived from Zhongshuang11 and No. 73290, of which the major QTL *qSN.A6* has been fine-mapped. In the current study, the near-isogenic lines (NILs) of *qSN.A7* were successfully developed by the successive backcross of F_1_ with Zhongshuang11. First, the effect of *qSN.A7* was validated by evaluating the SNPS of two types of homozygous NILs from BC_3_F_2_ population, which showed a significant difference of 2.23 on average. Then, *qSN.A7* was successfully fine-mapped from the original 4.237 to 1.389 Mb, using a BC_4_F_2_ segregating population of 2,551 individuals. To further clarify the regulatory mechanism of *qSN.A7*, the two types of homologous NILs were subjected to genetic and cytological analyses. The results showed that the difference in SNPS between the two homologous NILs was determined by the embryonic genotypic effect. Highly accordant with this, no significant difference was observed in ovule number per ovary, ovule fertility, fertilization rate, and pollen fertility between the two homologous NILs. Therefore, the regulatory mechanism of *qSN.A7* is completely different from the cloned *qSS.C9* and *qSN.A6*. These results will advance the understanding of SNPS and facilitate gene cloning and molecular breeding in *Brassica napus*.

## Introduction

Oilseed rape (*Brassica napus* L.) is one of the most important crops in the world in which production ranks eighth among all crops in 2018 (https://apps.fas.usda.gov/psdonline). As the world's second-largest oil crop, rapeseed accounts for about 20% of the world's total oil production (Basunanda et al., 2010; Friedt et al., 2018). Yield is an essential trait for the genetic improvement of most crops (Shi et al., 2009). In the past decades of years, the yield of rapeseed increased very slowly (annual average growth rate = 0.93%), in comparison with its fast improvement in other crops, such as soybean [average annual growth rate (AAGR) = 2.91%] and maize (AAGR = 1.49%) (https://apps.fas.usda.gov/psdonline). Yield is also the most complex trait, which is directly determined by its components and indirectly affected by various yield-related traits (Quijada et al., 2006; Shi et al., 2009; Zhao et al., 2016). Although the three components of rapeseed yield show varying degrees of negative correlation, the coefficients are usually small (Lu et al., 2011; Zhang et al., 2011; Cai et al., 2014; Shi et al., 2015). Therefore, rapeseed yield can be improved by the increase of its components. In rapeseed, seed yield is multiplicatively determined by its three components: silique number, seed number per silique, and seed weight (Clarkei and Simpson, 1978). The previous study showed that the seed number per silique is tightly correlated with seed yield (Raboanatahiry et al., 2018; Khan et al., 2018). In addition, the seed number per silique also showed a relatively high heritability, which makes it an important target for selection (Shi et al., 2009; Basunanda et al., 2010; Shi et al., 2015).

In the rapeseed germplasm resources, the seed number per silique shows a huge variation from about 5 to 35 (Chen et al., 2001). However, the underlying mechanisms have been rarely studied and poorly known. In a very early study, the X-ray technique was used to observe the development of silique and found that the failure of double fertilization is the main factor leading to a less seeds per silique (Pechan and Morgan, 1985). The cytological observation of Huashuang4, Zhongshuang11, and nine double haploid lines revealed that the difference of the seed number per silique in these lines was mainly due to the abortion of ovules (Li et al., 2014). Due to the complexity of seed number per silique, our previous study proposed the idea of decomposed it into four components: i.e., the number of ovules per ovary, the proportion of fertile ovules, the proportion of fertile ovules to be fertilized, the proportion of fertilized ovules to develop into seeds (Yang et al., 2017). The relative contributions of the four components were then studied using the representative four high-seed number per silique (SNPS) and five low-SNPS lines selected from a core association population (Li et al., 2019), which accounted for 30.7, 18.2, 7.1, and 43.9%, respectively (Yang et al., 2017).

The traditional genetic analysis on seed number per silique was concentrated on main plus polygene and additive-dominant-epistatic model as well as heritability and combining ability estimation. Li and Gu (1992) used the additive-dominant-epistatic model with six generation populations in four cross to analyze the heterosis and gene effects of several traits in rapeseed. The results showed that the additive effect was important for the seed number per silique. Radoev et al. (2008) used the NCII model with six rapeseed accessions to investigate the inheritance of seed number per silique in rapeseed, the results showed that this trait was governed by non-additive effects. Zhang et al. (2008) used the augmented NCII design with two high-SNPS as female parents and four low-SNPS lines as male parents to investigate the heterosis, combining ability, and genetic model of the seed number per silique, the results showed that both additive and non-additive genes governed this trait. Sabaghnia et al. (2010) used the additive main effects and multiplicative interaction (AMMI) model with a half diallel of nine rapeseed cultivars to quantitatively examine the genetic parameters for many traits in rapeseed, and concluded that seed number per silique was controlled by additive and non-additive gene effects. In addition, the seed number per silique has a strong heterosis and high heritability (Zhang et al., 2010; Shi et al., 2011). In our previous study, the representative four high-SNPS and five low-SNPS lines were subjected artificial self- and reciprocal cross-pollination experiment. The results showed that the seed number per silique of hybrid F_1_ was mainly determined by the general combining ability (63%) of the parents, followed by the special combining ability (37%) of parental combination, which was mainly determined by the additive effect, followed by the dominant effect (Yang et al., 2017).

Seed number per silique belongs to the seed traits, whose genetic model in theory includes the effects of four systems: embryo, endosperm, cytoplasm, and maternal genotype (Zhu, 1996). According to the formation and development of seeds, the seed traits should be determined by both maternal and offspring tissues (Mo, 1990). Early stage studies had shown that the seed number per silique was controlled by nuclear genes (Wei, 2000). The following research showed that the seed number per silique was also affected by maternal and cytoplasm effects (Li N. et al., 2015). In our previous study, the 2nGoCGm model was adopted to reciprocal crosses experiments between four high-SNPS and five low-SNPS lines. The results showed that the maternal, embryo, and cytoplasm genotype respectively explained 47.6, 35.2, and 7.5% of the genetic variance (Yang et al., 2017).

Up to now, nearly 100 quantitative trait loci (QTLs) of seed number per silique have been identified in rapeseed, which is a powerful illustration of the complex quantitative traits of multi-gene control in rapeseed, with a very complicated genetic basis (Quijada et al., 2006; Udall et al., 2006; Chen et al., 2007; Li et al., 2007; Radoev et al., 2008; Shi et al., 2009; Basunanda et al., 2010; Fan et al., 2010; Chen et al., 2011; Zhang et al., 2011; Yang et al., 2012; Cai et al., 2014; Qi et al., 2014). These QTLs are distributed on the A1, A2, A5, A7, A8, A9, C1, C2, C3, C4, C6, C7, and C9 chromosomes, respectively, explaining the phenotypic variance of 0.78–57.77%. Many studies (Chen et al., 2007; Basunanda et al., 2010; Zhang et al., 2010; Zhang et al., 2011; Yang et al., 2016) have shown that the additive and dominant effects were both significant for these QTL of seed number per silique. Of these, only two *qSN.A6* and *qSS.C9* were defined as major QTLs (Shi et al., 2015). Among them, *qSN.A6* was fine-mapped by our lab (Yang et al., 2016). To date, only *qSS.C9* has been cloned, which encodes a predicted small protein containing EST1 domain with 119 amino acids and regulate the formation of functional female gametophyte (Li N. et al., 2015 **).

In a previous study of our lab, a moderate-effect QTL-*qSN.A7* was detected using the F_2_/F_2:3_ population derived from two parental cultivars, Zhongshuang11 and No. 73290, which showed significant difference in seed number per silique (Shi et al., 2015). In this study, we try to fine-map this QTL and dissect its cytological mechanism, which lay a foundation for the further gene cloning. In detail, the main objectives of the current study were: 1) validation of the above-mentioned moderate-effect QTL *qSN.A7*; 2) fine-mapping of the target QTL *qSN.A7*; and 3) preliminary dissection of the regulatory mechanism of *qSN.A7*.

## Materials and Methods

### Construction of Near-Isogenic Lines for Target Quantitative Trait Locus

**Figure 1:**
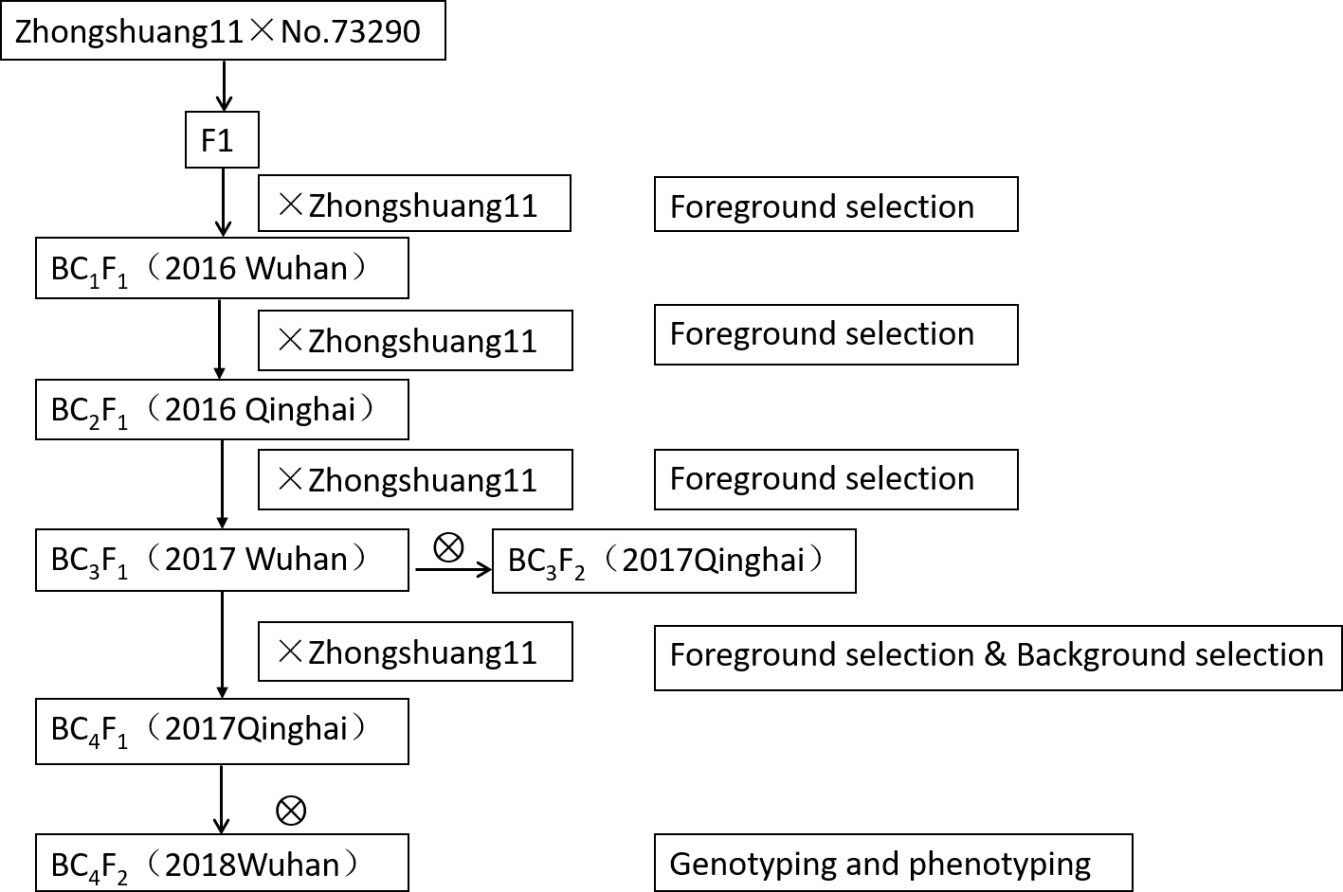
Procedure for the development of near-isogenic lines (NILs) population. The descriptions in the parenthesis following each generation of materials were the year and location.

The QTL near-isogenic lines (NILs) were constructed by successive backcrossing of F_1_ (between two parents Zhongshuang11 and No. 73290) with Zhongshuang11 for four times (**Figure 1**). In the process of NILs construction, the target segments were genotyped using the markers on both sides of the *qSN.A7*, and the individual plants with heterozygous status were screened.

The NILs at BC_3_F_1_ */*BC_4_F_1_ generation were used to for fore- and background analyses, and the individuals with heterozygous genotype at *qSN.A7* region and high recovery rate for background were selected to self-cross to produce BC_3_F_2_ */*BC_4_F_2_. The genotypes of the individual plants were identified, and the effects of the *qSN.A7* locus were determined by comparing the phenotypes of the two types of homozygous NILs.

### Development of Molecular Markers for the Target Quantitative Trait Locus

The corresponding genomic regions of the moderate-effect target QTL (*qSN.A7*) were identified by the alignment of the primer sequences of two flanking simple sequence repeat (SSR) markers (Niab043 and CNU339) with the genomic sequences of *B. napus* (Chalhoub et al., 2014). Bioinformatics softwares (BWA, SAMtools, GATA, etc.) were used to align and compare the genomic sequence of No. 73290 (re-sequencing) with the reference genome of zhongshuang11 (*de-novo* sequencing) to determine the location of SSR/InDel (Doyle et al., 2002; Huang et al., 2010; Jiao et al., 2012). The Primer 5 software was used to design primers on both sides of SSR/InDel sites (Untergasser et al., 2012). The designed primers were tested by electronic PCR against the reference genome to determine whether they were located between the target regions, and the specific primer with only one binding site were selected for synthesis. All of the newly developed SSR/InDel primers were tested in turn using two parents with polyacrylamide gel electrophoresis (PAGE) technique, of which specific and polymorphic primers were selected. The selected primers were validated using BnaZNRIL population, and the Zhongshuang11 genotypes were identified as A, No. 73290 genotype is identical to B, and the heterozygous genotype is identical to H. The JoinMap 4.0 software (http://www.kyazma.nl/index.php/mc.JoinMap) was adopted to construct the genetic linkage map using the newly developed markers and flanking markers Niab043 and CNU339 with a threshold for goodness-of-fit of ≤5, a recombination frequency of <0.4, and a minimum logarithm of odds (LOD) score of 2.0 (Voorrips, 2002; Van Ooijen, 2006; Mechanism and Agamospermy, 2010). All marker distances derived from the Kosambi function are expressed in centimorgan (cM).

### Genotypic Analyses

Leaf tissue was sampled from the individual plants of BC_1_F_1_, BC_2_F_1_, BC_3_F_1_, BC_3_F_2_, BC_4_F_1_, and BC_4_F_2_ populations (Saghai-Maroof et al., 1984). Genomic DNA was extracted using the cetyl trimethylammonium bromide (CTAB) method (Warude et al., 2003). PCR was performed in a volume of 20 µl containing 0.2 mM deoxynucleoside triphosphate (dNTP), 0.5 U Taq DNA polymerase, 75 ng of template DNA, 0.5 mM per primer, and 1 × PCR buffer (10 mM Tris pH 9.0, 5 mM KCl, and 1.5 mM MgCl_2_). DNA amplification was performed using the “touchdown” method, with the following temperature profile: initial denaturation at 94°C for 5 min; 34 cycles of 30 s at 94°C, 45 s at 56°C, 1 min at 72°C, and a final cycle of 5 min at 72°C. PCR products were separated on 6% polyacrylamide denaturing gels and visualized using the silver-staining method (Vos et al., 1995).

### Field Planting and Traits Measurement

The backcross populations at the different generations (BC_1_F_1_, BC_2_F_1_, BC_3_F_1_, BC_3_F_2_, BC_4_F_1_, and BC_4_F_2_) were alternatively planted in Wuhan and Qinghai for the consecutive 3 years from 2015 to 2018 (**Figure 1**). All populations and the two parents were planted by manual sowing, and the field management followed standard agriculture practices. Each row was 2-m long and 33.3-cm width, containing 18 plants. At maturity, the screened plants were harvested and air-dried at room temperature for approximately 2 weeks before testing. Seed number per silique was calculated as the average number of well-filled seeds from 30 well-developed siliques randomly sampled from the main branch.

For several recombinant types with only several plants, their self-crossed seeds were planted for offspring test (Yang et al., 2012).

### Identification of Female and/or Male Origin of *qSN.A7* Effect

To identify the female and male origin of the *qSN.A7* effect, a unique genetic-mating experiment was designed (Hua et al., 2012 **). For the genetic experiment, 10 pairs of plants were used for self- and cross-pollinated between NIL-Zhongshuang11 and NIL-No. 73290. The alternative branches on the same mother plant of NIL-Zhongshuang11 and NIL-No. 73290 were self- and cross-pollinated with each other, respectively (Li N. et al., 2015). Each self-/cross-pollination was repeated three times on the same plant, and pollinations were completed within 1 day. At maturity, the hand-pollinated siliques were harvested and threshed to measure the seed number per silique. Then multiple comparisons of seed number per silique were conducted among the hand-pollinated siliques of NIL-Zhongshuang11, NIL-No. 73290, and the reciprocal crosses (Li et al., 2020).

### Dissection of the Cytological Mechanism of *qSN.A7*

The exclusion method was used to clarify the cytological mechanism of *qSN.A7* in regulating the seed number per silique (evaluation of pollen vitality, pollen germination efficiency, pollen tube growth, ovule number, and embryo sac fertility). Both NIL-Zhongshuang11 and NIL-73290 were planted as three replicates.

For the microscopic observation of pollen, five individuals were randomly selected from each replicate. To test the viability of pollen, pollen of NIL-Zhongshuang11 and NIL-No. 73290 was collected from recently completely open flowers and stained with 1% acetocarmine (Marks, 1954). Then, the color and morphology of pollen were observed by a light fluorescence microscope (IX-71; Olympus, Tokyo, Japan). To determine the pollen germination efficiency *in vitro*, pollen of NIL-Zhongshuang11 and NIL-73290 was collected and evenly distributed in on a pollen germination medium (PGM) (10% sucrose, 0.005% H_3_BO_3_, 10 mM CaCl_2_, 0.05 mM KH_2_PO_4_, 6% PEG 4000) glass slide with 0.3% agar and then let sit at room temperature for 3 h, as described above (Schreiber and Dresselhaus, 2003). Then, the morphology of pollen was observed by a light fluorescence microscope (IX-71; Olympus, Tokyo, Japan). To determine the pollen germination efficiencies *in vivo* and the path of pollen tubes inside the pistil, pistils at one and three DAF were cut (ovary wall was removed) and fixed in 50% formalin acetic alcohol (FAA) (50% ethanol, 5% glacial acetic acid, 3.7% formaldehyde, v/v) for 24 h, softened in 8 M sodium hydroxide (NaOH) at 65°C for 3 h, washed with 50 mM K-phosphate buffer (pH = 7.5), and stained in 0.1% aniline blue (Schreiber and Dresselhaus, 2003). Then a light fluorescence microscope (IX-71; Olympus, Tokyo, Japan) was used to observe the stained pistils and pollen tubes.

For the microscopic observation of ovule, the flower buds of different lengths were collected. These flower buds come from five randomly selected plants for each replicate. To investigate the number of ovules per ovary before flowering, we collected pistils at different developmental stages. The buds of 1 day before flowering (DBF) were collected and fixed in 50% FAA solution, dehydrated through an ethanol series of 75, 85, 95, and 100% (v/v), then put into the solution with a 1:1 volume ratio of methyl salicylate to alcohol for 40 min (Smyth et al., 1990). Hereafter, it was store in 100% methyl salicylate for more than 24 h and observed using a light fluorescence microscope (IX-71; Olympus, Tokyo, Japan).

To investigate the embryo sac before flowering, we collected ovules at different developmental stages. These flower buds come from five randomly selected plants for each replicate. The buds of one DBF were collected and fixed in 50% FAA solution more than 24 h. Wash with sterilized water three times, and then soak with 6 mol/L NaOH more than 12 h. Wash with sterilized water three times, then soak with 0.14 mol/L K_2_HPO_4_ (pH = 8.2) and 0.1% water-soluble aniline blue for more than 24 h and observed using a light fluorescence microscope (IX-71; Olympus, Tokyo, Japan) (Skinner and Sundaresan, 2018).

## Results

### Validation of *qSN.A7*

To validate the effect of *qSN.A7*, NIL populations were constructed by successive crossing of F_1_ with the elite cultivar Zhongshuang11, because it has high resistance to disease and lodging as well as the genomic information.

To determine the effect of *qSN.A7*, the NIL population at BC_3_F_2_ generation was used for analysis. A total of 150 BC_3_F_2_ plants were genotyped using two SSR markers (Niab043 and CNU339) flanking the target region. Of which, 38 and 31 individuals were homozygous for Zhongshuang11 and No. 73290 respectively, whereas the other 72 plants were heterozygous. We harvested and measured the seed number per silique of the three types of non-recombination plants. The results showed that the average seed number per silique of NIL-zhongshuang11 (18.86 ± 0.50) was not significantly different (*P =* 2.95E−01) with the NIL-heterozygote (17.64 ± 0.73), meanwhile the average seed number per silique of the NIL-Zhongshang11 (*P =* 8.65E−03) and NIL-heterozygote (*P =* 2.84E−02) were significantly more than that (16.63 ± 0.33) for NIL-No. 73290 (**Table 1**).

**Table 1:** Comparison of seed number per silique for three types of non-recombinant near-isogenic lines (NILs) screened from BC_3_F_2_ population.

Genotype	Number of individuals	Niab043	CNU339	Seed number per silique
NIL-Zhongshuang11	38	A	A	18.86 ± 0.50a
NIL-heterozygous	72	H	H	17.64 ± 0.73a
NIL-No. 73290	31	B	B	16.63 ± 0.33b

Genotype A, B, and H represent homologous alleles from Zhongshuang11 and No. 73290 as well as heterozygous alleles, respectively. The letters following the phenotypic values represent the significance of difference in multiple comparisons.

This showed that target QTL-*qSN.A7* had a significant effect for seed number per silique, which was about 2.23 at BC_3_F_2_ generation.

### Fine-Mapping of *qSN.A7*

#### Development of InDel Markers

According to the results of primary-mapping, the *qSN.A7* was located in in the 15,885–20,122 kb region of A7 pseudo-chromosome of the reference genome Darmor_V8.1, between SSR markers Niab043 and CNU339. A total of 273 InDel loci were found within the target region based on the genomic data of the two parents Zhongshuang11 (*de-novo* sequencing) (Sun et al., 2017) and No. 73290 (re-sequencing) (Huang et al., 2013). A total of 31 primer-pairs were designed flanking these InDel loci (**Supplementary Table 1**), which were named Ni201 to Ni210 and BnID301 to BnID321.

Subsequently, these newly synthesized primer-pairs were tested using two parents, of which 14 were polymorphic and single locus. Then, they were genotyped using the BnaZNRIL population, and four InDel markers were found to be linked within the abovementioned two SSR markers flanking *qSN.A7* (**Table 2**). These four successfully developed InDel markers were BnID306, Ni201, BnID320, and Ni206, which were used to further narrow the region of *qSN.A7*.

**Table 2:** Information on markers used for the fine-mapping of *qSN.A7*.

Marker	Forward_primer	Reverse_primer	Chr_Start	Chr_End
Niab043	CCATTCGAGGTGGTCGTAAA	AGAAAACGGACCTCGATTCA	19404296	19404580
BnID306	CATTGTACAACCAAAGATTATATCCC	TTTTATCCGTTTAGCAAAAGCTAGT	19932838	19932981
Ni201	AAACGCAAGTGCTATGTCCC	CCACGGAAAACTTGTAACGG	22685861	22686103
BnID320	TCTGGCCAAAACATATATGGAGTA	GTTCCTTTTGAGTTCGTTTGAGTT	24162107	24162216
Ni206	TGTATACACAGGCAAAGCAGC	CAAAGCTCACGTTCCTGGAT	24358131	24358316

#### Construction of BC_4_F_2_ Population for Fine-Mapping

First, the 1536 BC_4_F_1_ individuals were genotyped using the two SSR markers (i.e., foreground selection) flanking *qSN.A7*, of which 85 heterozygous plants were screened. Then, they were subjected to background selection using a total of 80 SSR/SNP markers that are evenly distributed on the 19 linkage groups, including two SSR markers flanking the major SNPS QTL-*qSN.A6*. The background proportions of these plants ranged from 86.7 to 98.6%, and several individuals with >95% were self-crossed to produce BC_4_F_2_. Finally, a BC_4_F_2_ population of 2551 individuals was constructed to conduct fine mapping of *qSN.A7*.

**Figure 2:**
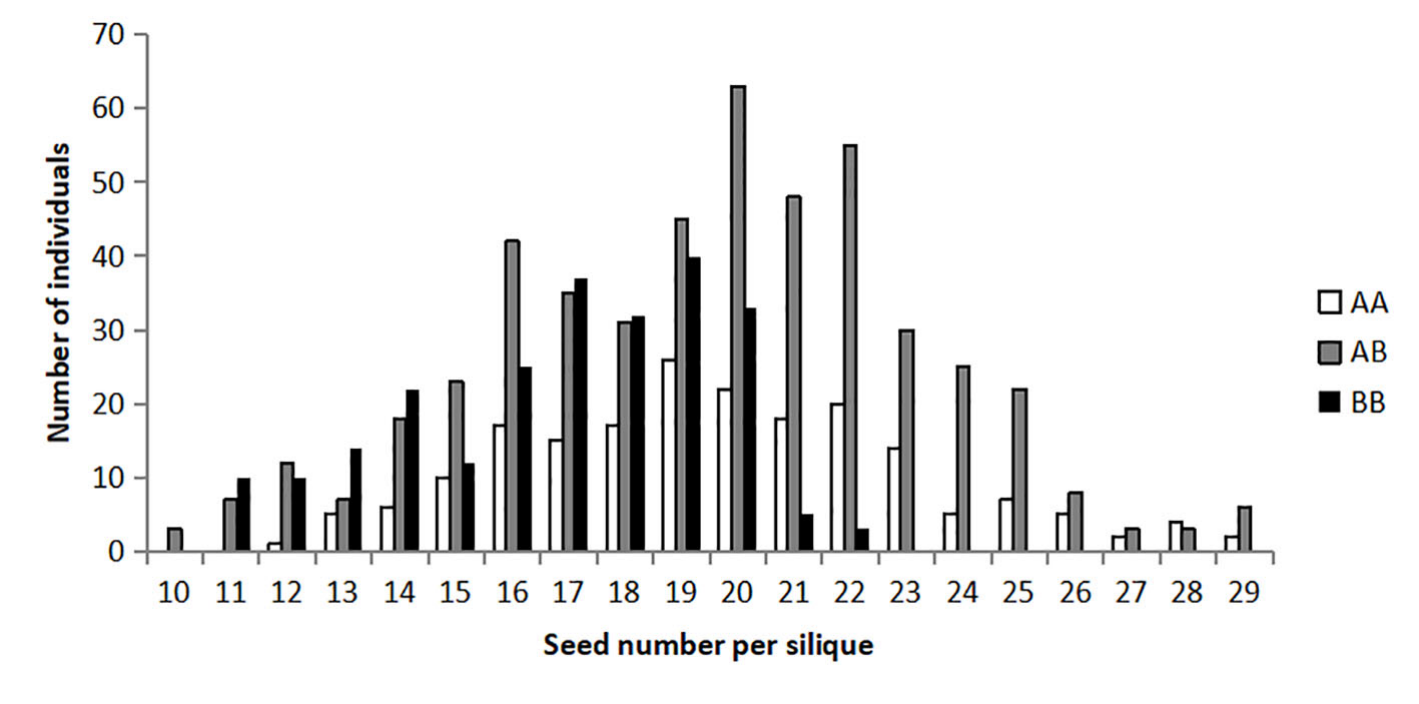
Frequency distribution of seed number per silique in the BC_4_F_2_ population. The horizontal axis represents the trait value of seed number per silique. The vertical axis represents the number of individuals. The three types of genotype are represented by the three column colors, as shown in the legend.

As expected, the frequency distribution of seed number per silique of the BC_4_F_2_ population deviated from the normal distribution (D = 0.03, *P <*0.01) and appeared to be a bimodal distribution (**Figure 2**). The Chi-square analysis on the phenotypic values of seed number per silique in the BC_4_F_2_ population showed that it was basically accordant with a segregation ratio of 3:1 (χ^2^ = 0.1051, *P =* 7.46E−01) distribution, which indicated that one Mendelian locus controlled this trait in this NIL population.

In the BC_4_F_2_ population, the numbers of three types of non-recombinant individuals (211:481:225), homozygous for the Zhongshuang11 allele (AA), heterozygous (AB), and homozygous for the No. 73290 allele (BB), showed an expected ratio of 1:2:1 (χ^2^ = 2.6358, *P =* 2.68E−01), indicating an absence of distorted segregation. The seed number per silique of the BC_4_F_2_ plants with the AA genotype (19.47 ± 1.31) was slightly higher (*P =* 5.10E−22) than that for the BB genotype (16.41 ± 1.54), and the heterozygotes (19.51 ± 0.17) were much higher (*P =* 3.36E−30) than for the BB genotype (**Table 3**). It should be noted that, there were no significant difference (*P =* 4.29E−01) between the AA genotype (19.47 ± 1.31) and the heterozygotes (19.51 ± 0.17). These results suggested that the favorable allele of *qSN.A7* was from Zhongshuang11, and the mode-of-inheritance of *qSN.A7* was full-dominance.

**Table 3:** Comparison of seed number per silique for several types of homozygous near-isogenic lines (NILs) screened from BC_4_F_2_ population.

Types	Number of individuals	Five markers within *qSN.A7* interval					Seed number per silique	
		Niab043	BnID306	Ni201	BnID320	Ni206	Mean ± SD	*P*
Non-recombinant	30	A	A	A	A	A	19.47 ± 1.31	CK
	30	B	B	B	B	B	16.41 ± 1.54	<0.0001
Recombinant	35	B	A	A	A	A	19.53 ± 1.71	0.6655
	34	B	B	A	A	A	19.38 ± 2.24	0.6763
	40	B	B	B	A	A	18.97 ± 1.97	0.8239
	32	B	B	B	B	A	16.49 ± 1.70	<0.0001
	36	A	B	B	B	B	15.94 ± 0.83	<0.0001
	38	A	A	B	B	B	16.73 ± 2.21	<0.0001
	30	A	A	A	B	B	19.45 ± 0.36	0.5689
	37	A	A	A	A	B	20.27 ± 1.61	0.8382

Genotype A and B represented homologous alleles from Zhongshuang11 and No. 73290, respectively. The letter following the phenotypic values represented the significance of difference in multiple comparisons.

#### Further Narrowing the Target Region of *qSN.A7*

Five evenly distributed co-dominant SSR and InDel markers within the 4.24 Mb interval of *qSN.A7* were used for genotyping the 282 homozygous single-exchange individuals. According to the recombinant point between the five markers, these single-exchange plants can be classified into eight types (**Table 3**). Obviously, the eight types of recombination were naturally grouped into two groups based on the comparison of their seed number per silique with the recurrent parent Zhongshuang11 (19.47 ± 1.31). The first group included four types whose seed number per silique (18.97 ± 1.97 to 20.27 ± 1.61) was not significantly different from Zhongshuang11. The seed number per silique (15.94 ± 0.83 to 16.73 ± 2.21) of the other groups was significantly lower than that for Zhongshuang11. All three types of recombination have a common introgression fragment between markers Ni201 and BnID320. According to this, the genomic region containing the *qSN.A7* was delimited to an interval of approximately 1.389 Mb.

### Genetic and Cytological Mechanism of *qSN.A7*

To identify the key factor responsible for the seed number per silique difference between NIL-Zhongshang11 and NIL-No. 73290, the following genetic and cytological experiments were performed.

#### Genetic Analysis of *qSN.A7*

**Figure 3:**
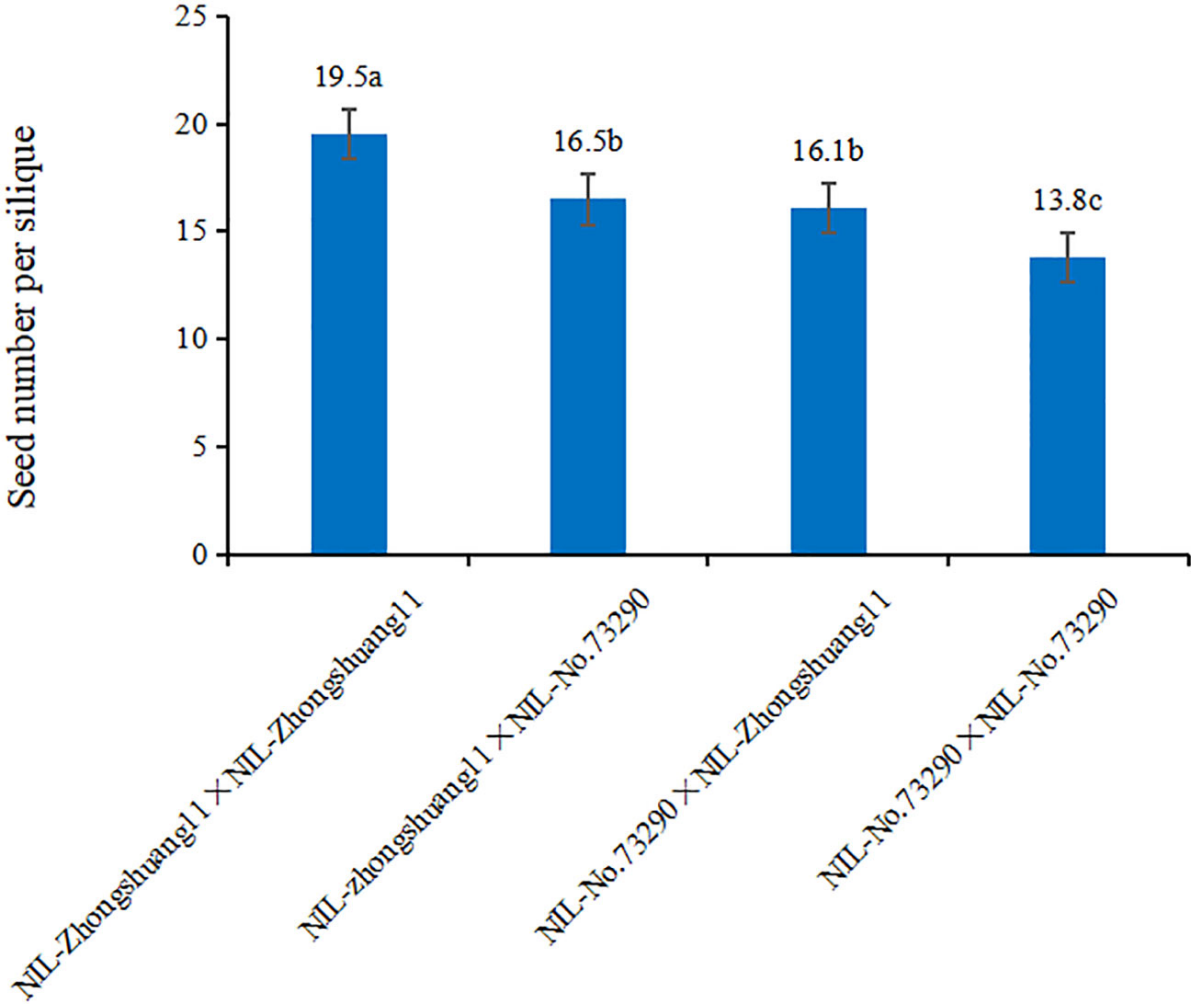
Comparison of the seed number per silique between the self- and cross-pollinations. The vertical and horizontal axes represent the seed number per silique of the different combinations between NIL-Zhongshuang11 and NIL-No. 73290, respectively. The letter following the numerals represent the significance of difference in multiple comparisons.

To identify the male or female origin of the *qSN.A7* effect, self- and cross-pollination were performed by hand between NIL-Zhongshang11 and NIL-No. 73290. The results showed that the seed number per silique of the reciprocally cross-pollinated siliques between NIL-Zhongshang11 and NIL-No. 73290 were not significantly different (**Figure 3**). At the same time, the seed number per silique of self-crossing of NIL-Zhongshang11 was significantly higher than that for cross-pollination, which was also significantly higher than that for self-crossing of NIL-No.73290. These results showed that the effect of *qSN.A7* was determined by embryonic genotype, which are related to both male and female parents.

#### Cytological Analysis of *qSN.A7*

**Figure 4:**
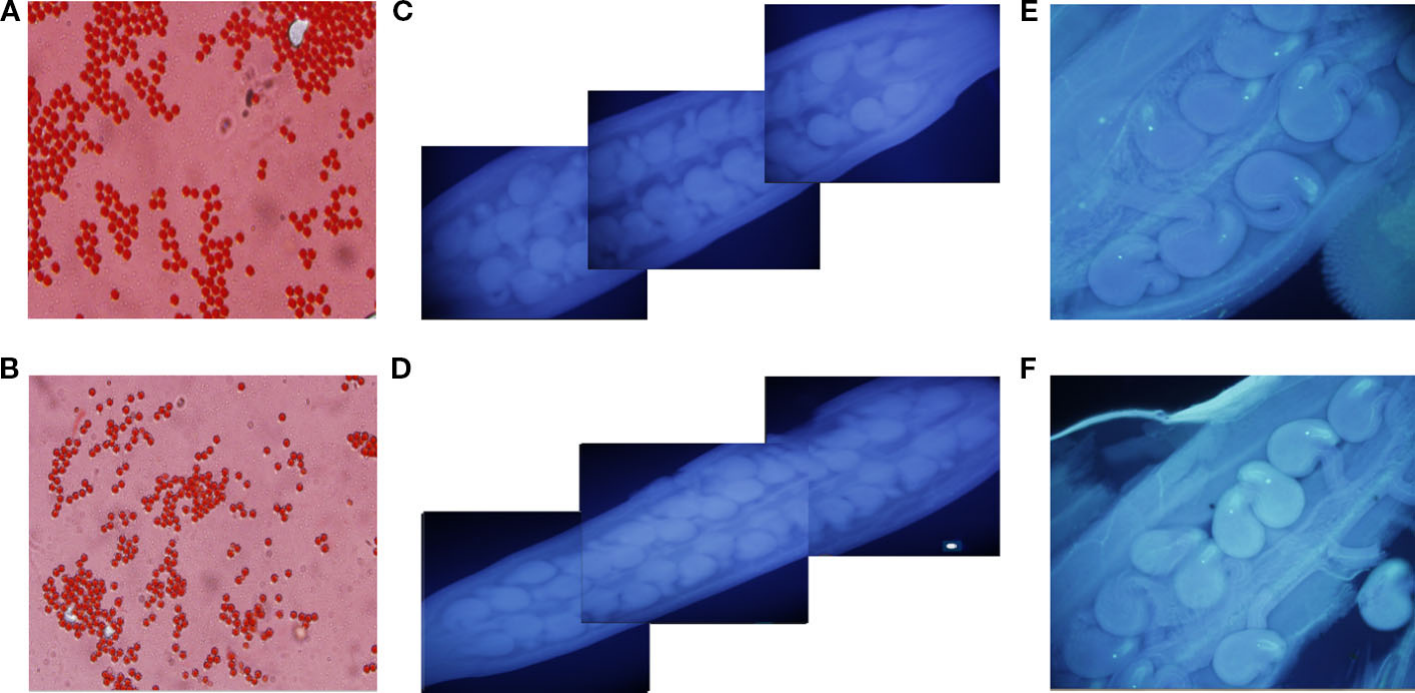
Cytological observation of the number of ovules, pollen viability, ovule fertility. Pollen of both lines appears to be fertile **(A**, **B)**. Pollen germination *in vitro* of both NIL-Zhongshuang11 and NIL-No. 73290 is normal **(C**, **D)**. There is no difference in ovule fertility of both Zhongshuang11 and NIL-No. 73290 is normal **(E**, **F)**.

First, to identify whether the seed number per silique difference between NIL-Zhongshuang11 and NIL-No. 73290 was related to pollen, the main characteristics (including pollen vitality, pollen germination efficiency, pollen tube growth) reflecting the quality and quantity of pollen for both lines were checked. The results showed that there was no significant difference between two NILs (**Figures 4A, B**). Secondly, to determine whether the seed number per silique difference between Zhongshuang11 and NIL-No. 73290 was related to the fertilization, the process of pollen tube elongation was observed, and no significant difference was found. Then we investigated whether the seed number per silique difference between Zhongshuang11 and NIL-No. 73290 was related to ovules. The ovules number and embryo sac fertility was observed before flowering. The results show that no significant (*P > *0.1) difference in both ovules number (**Table 4**) and embryo sac fertility between the two NILs (**Figures 4 C–F**).

**Table 4:** Comparison of ovule number per ovary sampled from different sizes of buds before flowering.

	2 mm	3 mm	4 mm	5 mm	6 mm	7 mm	8 mm
**NIL-No. 73290**	26.71± 0.2	25.44 ± 0.6	26.52± 1.9	25.81± 0.7	26.44± 1.6	27.64± 0.8	29.33± 0.5
**NIL-Zhongshuang11**	27.56± 0.6	26.17± 1.6	27.68± 0.7	27.00± 1.3	27.85± 1.1	28.79 ± 0.8	27.4± 1.5
**Pt-test**	4.74E−1	3.99E−1	1.30E−1	1.80E−1	2.04E−1	2.21E−1	4.27E−1

Previous studies have shown that the number of ovules per ovary, the proportion of fertile ovules, the proportion of fertile ovules to be fertilized, the proportion of fertilized ovules all determined the seed number per silique (Li et al., 2014; Li S. et al., 2015; Yang et al., 2017). The above experimental results showed that the number of ovules per ovary and the proportion of fertile ovules had no difference between NIL-Zhongshang11 and NIL-No. 73290. Therefore, the effect of *qSN.A7* should be related to the development of fertilized ovule, which was accordant with the conclusion of genetic analysis (controlled by the embryonic genotype).

## Discussion

### Verification of Quantitative Trait Locus Effect

Because the QTL with large effects are generally stable in different environments, researchers prefer to select major QTL for fine mapping and cloning (Venuprasad et al., 2012; Bonneau et al., 2013; Dixit et al., 2015). Some studies have shown that QTL with small effect are inconsistent in different environments (Xing et al., 2002), whereas some other studies have shown that small-effect QTL can also be stable (Wei et al., 2010; Chen et al., 2014). Since there is no report on the fine-mapping and cloning of non-major QTLs in rapeseed, this study is the first to attempt to fine positioning the moderate-effect QTL in rapeseed.

Our lab previously identified two repeatable QTLs for seed number per silique using the F_2_/F_2:3_ populations constructed from two sequenced cultivars Zhongshuang11 and No. 73290, namely *qSN.A6* and *qSN.A7* (Shi et al., 2015). The additive effect and phenotypic variance explained by the target QTL *qSN.A7* were 0.86 and 15.8%, respectively, which was a moderate-effect QTL. However, *qSN.A7* has not been detected in the recombinant inbred line population derived from the same parents (Yang et al., 2016). In addition, the effects of some QTLs may become non-significant or disappear in the NILs background (Yang et al., 2010; Ravi et al., 2011). Therefore, it is very necessary to verify its effect using NILs before the subsequent map-based cloning.

Comparison of the seed number per silique between two types of homologous BC_3_F_2_ NILs showed that the difference was significant, which indicates that *qSN.A7* is real and effective, and can be used for subsequent fine-mapping and cloning. In addition, the additive effect of *qSN.A7* was 1.12 in the NILs background, which is a little larger than its corresponding value in F_2_ background. In fact, the additive effect of most mendelized QTLs are also larger than their corresponding value in primary mapping. However, the additive effects of *qSN.A7* in both the primary and fine mapping population were all lower than *qSN.A6* (Shi et al., 2015) and *qSS.C9* (Li S. et al., 2015 **), which represents a challenge in map-based cloning of moderate effect QTLs.

### Fine Mapping of the Quantitative Trait Locus for Seed Number Per Silique

To the present, about 100 QTLs for seed number per silique have been located in rapeseed, which are widely distributed on 17 linkage groups, except for A6 and C9 (Li S. et al., 2015; Yang et al., 2016). However, the major QTL is very few, only on several linkage groups including A1, A6, C1, and C9 (Shi et al., 2009; Chen et al., 2011; Li et al., 2014; Qi et al., 2014).

Generally, the major QTLs have been the focus of fine positioning and gene cloning. For the seed number per silique in rapeseed, only two major QTL have been successfully fine-mapped (Zhang et al., 2012; Yang et al., 2016) and only one has been furtherly cloned (Li N. et al., 2015 **). However, compared with the located major QTLs, the moderate- and small-effect QTLs are much more and widely distributed in the genome (Tanskely, 1993; Mackay, 2009; Huang et al., 2013). In recent years, moderate- and small-effect QTLs had gradually gained more attention, but these studies were mainly concentrated in rice (Chen et al., 2014; Zhang et al., 2015).

In this study, the interval of moderate effect QTL-*qSN.A7* was successfully narrowed by a BC_4_F_2_ NIL population. This has outstanding reference value for the research on the large number of moderate- and small-effect QTLs in rapeseed.

### The Regulatory Mechanism of the Seed Number Per Silique in Rapeseed

The previous studies have investigated the regulatory mechanisms of seed number per silique difference between the different cultivars, through cytological observation using several techniques. The results showed that ovule number and fertility, double fertilization and seed development all affect the final seed number per silique, although their relative contributions might differ in different cultivars (Li et al., 2014; Li S. et al., 2015; Yang et al., 2017).

To the present, in the single gene/locus level, only two have been characterized. The *qSS*.C9 determines ovule fertility by regulating meiosis of megaspore mother cells (Li N. et al., 2015). Our previous study showed that *qSN.A6* locus also affected ovule fertility by regulating cellularization of the embryo sac (Yang et al., 2017).

In the current study, through a systematically genetic analysis and cytological observation, our results showed that *qSN.A7* is controlled by embryonic genotype probably by regulating the development of fertilized ovule. Therefore, the regulatory mechanism of *qSN.A7* is completely different with the previously reported two QTLs *qSS*.C9 and *qSN*.A6 those maternally control seed number by regulating ovule fertility.

## Data Availability Statement

All datasets generated and analyzed for this study are included and cited in the article/**Supplementary Material**.

## Author Contributions

JS and HW designed the experiments. JPZ, GL and XW provided the research materials. YZ and JJZ performed the fine-mapping and genetic hybridization experiments. YZ and JY performed cytological experiments. YZ and JY analyzed the data. YZ, and XZ wrote the manuscript.

## Funding

This research was supported by the National Key Research and Development Program (2018YFD0100601), the Natural Science Foundation (31101181), Wuhan Youth Science and Technology Morning Project (2017050304010286), the Rapeseed Industry Technology System (CARS-13), the Agricultural Science and Technology Innovation Project (CAAS-ASTIP-2013-OCRI), the Core Research Budget of the Non-profit Governmental Research Institution (1610172017001).

## Conflict of Interest

The authors declare that the research was conducted in the absence of any commercial or financial relationships that could be construed as a potential conflict of interest.

## Supplementary Material

The Supplementary Material for this article can be found online at: https://www.frontiersin.org/articles/10.3389/fpls.2020.00068/full#supplementary-material

Supplementary Table 1 Detailed information of Indel primers in target area.
