# Supplementary material for: Validation and Characterization of a Seed Number Per Silique Quantitative Trait Locus qSN.A7 in Rapeseed (Brassica napus L.)
Source: Front Plant Sci. 2020 Feb 21;11:68. doi: 10.3389/fpls.2020.00068 (PMC7047150; doi:10.3389/fpls.2020.00068)
Supplement: Supplementary Table 1 — Detailed information of Indel primers in target area. [file DataSheet_1.pdf]

| Primer name | Forward | Reverse |
|---|---|---|
| Ni201 | AAACGCAAGTGCTATGTCCC | CCACGGAAAACTTGTAACGG |
| Ni202 | CGTGCTCACACAATTGTCATT | CAGATGCTCATGTTGAACGC |
| Ni203 | CGTGCTCACACAATTGTCATT | CAGATGCTCATGTTGAACGC |
| Ni204 | CGAAAGACGTGACAGACAGC | GCCCGTTTGGGATTTTTAAT |
| Ni205 | TGTGTGACACTCCTTTCGTCA | CGTGACGCTGTTCTTAGTGTTC |
| Ni206 | TGTATACACAGGCAAAGCAGC | CAAAGCTCACGTTCCTGGAT |
| Ni207 | CTGAACCTCATCCTCTTCGC | AAGGAGAGAGACGCCATGAA |
| Ni208 | TGATGGGCACAGTTGATGTT | TACCAATGCTTCTGCCTTCC |
| Ni209 | CATTGGTATGGGTTTCTCCG | TGCCTCCTACAAAACAGCAA |
| BnID301 | CTTCCACAAGCATGAGACTTTCTA | AACAGAATCAAAAATAAGCAAGGC |
| BnID302 | TGCATTACGGTAAAAGTTTTTCAT | AAATAGGCAAAATTGAGAAAAGA |
| BnID303 | CAGAAGCCAGAAGGTATCAAGAA | AAAAAGAAAAATCCATCTTTGCAG |
| BnID304 | AAATTATATGTTTTGCCAGTTGCC | TAGTTATTCTGAGCGATTGTGACC |
| BnID305 | GCAACAAACACCATGTAAAACAA | TTTACGTTCTTTTTACTGACAGCG |
| BnID306 | ATTGTACAACCAAAGATTATATCC | TTTTATCCGTTTAGCAAAAGCTAGT |
| BnID307 | AACTGCTTGTACAGAAAACTGACC | ACAAAATGTATCGTGCTTCAAGAG |
| BnID308 | ACATCTCATCTGACCGAATAAACA | AGAAGCCAAGAAGGGTAAACCTAT |
| BnID309 | GGATGAGGTTCACAACAAATCAT | AAGTTGGGATATGAGAGAGGAGTG |
| BnID310 | ATGTGGGTGTGTTTGTTTATGCTA | AAAATGCTCTACTGAGCTAACGGT |
| BnID311 | ATTCTGACCAAGAGATGTACACGA | ACTCTCAGAATTCAAAATGTTCCC |
| BnID312 | TGTTGCATTACTTTAGGGAGCATA | TTTTTCCACAGTTTTGTTCTTCTG |
| BnID313 | AGGTTGCAAACTTCTACCAAGAAC | TATTTTGATGAGACCACTCACTCC |
| BnID314 | TTGTTGTTGTTGTACATTTTTGGT | CAAACAATATCATTGTGATACTTC |
| BnID315 | ATGAAGATATTGTAATTCGGCCA | CTTTCGATTTTGACGGTTTAGATT |
| BnID316 | TACCACATGCAGCACATTAACATA | GCAACCAGGATTAACAGCACTAAT |
| BnID317 | TTGAATAACTTGTTTCCCTCATCA | AACAAAGCAAGAATGGGAAGTTAC |
| BnID318 | TTTTTCTGGATAGAGCACTGTTGT | CCACCTAGAGTCACGAAAATGTAA |
| BnID319 | AGGCAAAGATTGAGTAATTTTGGA | TCACTTCTACGTACCCAAGTCTGA |
| BnID320 | TCTGGCCAAAACATATATGGAGTA | GTTCCTTTTGAGTTCGTTTGAGTT |
| BnID321 | AACTGTACCGATGAGGTTTTATGC | GCTTTCTTATGGTTAGCTGACTCG |

| Chr_name | Start | End |
|---|---|---|
| A07 | 22685861 | 22686103 |
| A07 | 24034026 | 24034287 |
| A07 | 24034026 | 24034287 |
| A07 | 24090098 | 24090234 |
| A07 | 24119167 | 24119442 |
| A07 | 24358131 | 24358316 |
| A07 | 24388367 | 24388579 |
| A07 | 38045246 | 38045375 |
| A07 | 24511774 | 24512054 |
| A07 | 19473469 | 19473591 |
| A07 | 19634099 | 19634277 |
| A07 | 19721677 | 19721807 |
| A07 | 19769067 | 19769192 |
| A07 | 19879393 | 19879554 |
| A07 | 19932838 | 19932981 |
| A07 | 20867687 | 20867845 |
| A07 | 21051817 | 21051944 |
| A07 | 21290149 | 21290277 |
| A07 | 22232861 | 22233017 |
| A07 | 22422408 | 22422550 |
| A07 | 22656598 | 22656749 |
| A07 | 22834818 | 22834957 |
| A07 | 22868943 | 22869079 |
| A07 | 23432760 | 23432862 |
| A07 | 23495640 | 23495757 |
| A07 | 23685537 | 23685668 |
| A07 | 23755908 | 23756052 |
| A07 | 23954883 | 23954965 |
| A07 | 24162107 | 24162216 |
| A07 | ######## | 24268486 |
